# Evaluation of pandemic potential of the genotype 4 (G4) swine influenza virus using ex vivo and in vitro cultures of the human respiratory tract

**DOI:** 10.1099/jgv.0.002133

**Published:** 2025-07-31

**Authors:** Jenny C.M. Chan, Rachel H.H. Ching, Hermione H.M. Kock, Teng Long, John M. Nicholls, J.S. Malik Peiris, Kenrie P.Y. Hui, Michael C.W. Chan

**Affiliations:** 1 School of Public Health, LKS Faculty of Medicine, The University of Hong Kong, Hong Kong SAR, PR China; 2 Centre for Immunology and Infection (C2i), Hong Kong Science Park, Hong Kong SAR, PR China; 3 Department of Pathology, School of Clinical Medicine, LKS Faculty of Medicine, The University of Hong Kong, Queen Mary Hospital, Hong Kong SAR, PR China

**Keywords:** *ex vivo*, influenza, innate host responses, risk assessment, swine influenza, tropism

## Abstract

Recent studies have reported a genotype 4 (G4) reassortant Eurasian avian-like (EA) H1N1 virus in swine, demonstrating a potential pandemic threat in humans. Here, we have compared the tropism, replication competence and pro-inflammatory cytokine and chemokine induction of the two G4 EA H1N1 strains in parallel with 2009 pandemic H1N1 (H1N1/pdm/09) and A/Quail/HK/G1/1997 H9N2 (G1) using *ex vivo* culture of the human respiratory tract and *in vitro* culture of human peripheral blood-derived macrophages. Our results showed that G4 strains could replicate in *ex vivo* cultures of human lung and bronchus with a similar replication competence to H1N1/pdm/09. The cytokine induction levels of G4 were similar to H1N1/pdm/09 in macrophages. Taken together, we could extrapolate that the G4 EA H1N1 swine influenza may pose a notable public health threat towards human and should not underestimate this threat.

## Introduction

Recent studies have reported a genotype 4 (G4) reassortant Eurasian avian-like (EA) H1N1 virus in swine, which was being comprised with the 2009 pandemic and triple reassortant (TR) derived internal gene and has become solely predominant in the swine population in China since 2016 [1]. They have reported that the G4 H1N1 strain can replicate effectively in human bronchial epithelial cells and alveolar epithelial cells and has exhibited efficient infectivity and transmissibility in ferrets, showing an increase in replication competence and pathogenicity. Documented human cases of G4-like EA H1N1 virus in China and other parts of the world have illustrated the potential possibility of the strain to transmit from swine to human [2,6], indicating the likelihood to cause more severe disease, and may raise concerns in the human population.

Influenza A is a highly infectious pathogen that can infect a range of species, including but not limited to human, swine and avian species. It is well documented that pigs are an intermediate host, which expressed both α-2,3- and α-2,6-linked sialic acids, and could serve as a mixing vessel to generate new influenza viruses. The expression of both sialic acids would allow swine, avian and human influenzas to bind to and potentially increase the chance for reassortment to take place, resulting in the generation of progeny viruses. While China has been characterized as a varied ecosystem of swine influenza viruses, with the classical lineage, North American TR lineage and EA lineage are known to co-circulate [7], favouring the reassortant event to take place. However, the EA H1N1 swine influenza viruses have gradually replaced other lineages to become the most dominant lineage in China since the first identification of this virus in Hong Kong in 2001 [7,8]. By lineage classification, it has been reported that six genotypes, namely, G1 to G6, were found in EA H1N1 viruses based on the surveillance of pigs in China from 2011 to 2018. G1 is being defined as the original EA H1N1 lineage, was the predominantly circulating lineage from 2011 to 2013 and disappeared after. G2, G3 and G6 reassortant EA viruses have temporarily appeared from 2011 to 2015, whereas G4 and G5 reassortant EA viruses that emerged in 2013 have later become the dominant strains. Other than the circulating swine influenza, the human pandemic strain H1N1/pdm/09 has spread back into pig herds after 2009. This has resulted in a reassortant between the swine EA H1N1 and H1N1/pdm/09 influenza virus that has been sporadically detected in pig farms [9,11].

In this study, we have compared the tropism and replication competence [11] of the G4 EA H1N1 swine influenza in parallel with 2009 pandemic H1N1 (H1N1/pdm/09) and A/Quail/HK/G1/1997 H9N2 (G1) using a highly physiologically relevant human model of *ex vivo* explant cultures of human lung and bronchus. We have also investigated the cytokine and chemokine induction of the G4 EA H1N1 swine influenza using peripheral blood monocyte-derived macrophages and compared it with H1N1/pdm/09 and G1. The H1N1/pdm/09 strain was used in this study as a hallmark case that could demonstrate a successful human influenza virus of a swine origin that results in a pandemic and causes mild disease in 2009 [12,14]. Meanwhile, the high cytokine-inducing avian influenza G1 [15] was used as an alternative control to represent the high cytokine-inducing influenza strain, which shares commonality in internal genes with the high pathogenicity avian influenza H5N1/1997 [16,18]. Consequently, both could act as a good reference for the risk assessment of infectivity, transmissibility and pathogenicity of the G4 strains.

## Methods

### Virus propagation and strains used

Two strains of G4 EA H1N1 influenza viruses, A/Swine/Hong Kong/NS13/2014 and A/Swine/Hong Kong/NS2885/2014 (abbreviated as SW13 and SW2885, respectively), were isolated from swine nasal swab collected from Sheung Shui Slaughterhouse, Hong Kong. Two strains of human influenza A viruses, the pandemic influenza virus A/Hong Kong/415742/2009 (abbreviated as H1N1/pdm/09) isolated from a patient in Hong Kong and high cytokine-inducing H9N2 avian influenza A/Quali/Hong Kong/G1/1997 (abbreviated as G1) isolated from quail, were used in the study to risk assess the two G4 EA H1N1 influenza viruses.

Virus propagation was performed using the Madin–Darby canine kidney (MDCK) cells with limited passages. Virus stocks were prepared in MDCK cells, and viral titres were determined by plaque assay and TCID_50_ assay as previously described [19].

### *Ex vivo* cultures and infection

Human lung and bronchial tissues were removed as part of a routine clinical care from patients undergoing surgical resection in Hong Kong. The samples were taken from non-tumour regions, which were assessed by the pathologist. Infection of *ex vivo* cultures was performed as previously described [19] and was performed in at least three independent donors. In brief, bronchial and lung tissues were infected with each virus with 100 µl of 1e6 TCID_50_ per millilitre for 1 h at 37 °C and were subsequently washed with 1× PBS for three times to remove any unbound virus. The lung tissues were placed directly in culture medium (F12-K with 100 units/ml penicillin and 100 µg ml^−1^ streptomycin), and the bronchial tissues were placed on sterile surgical sponges to establish an air–liquid interface condition. Tissues that were inoculated with medium only served as a negative control. Viral replication efficiency was assessed by TCID_50_ assay titrating the supernatants collected at 1, 24, 48 and 72 h post-infection (hpi) using MDCK cells. Bronchial and lung tissues were then fixed at 72 hpi in 10% formalin and further processed for immunohistochemistry and immunofluorescence staining.

### *In vitro* human macrophage isolation and infection

Isolation of human peripheral monocytes and generation of primary macrophages were performed as previously described [20]. In brief, healthy peripheral blood leukocytes provided by the Hong Kong Red Cross Blood Transfusion Service were isolated by centrifugation on Ficoll-Paque density gradient and further purified by adhesion. The primary macrophages were seeded onto tissue culture plates and maintained with RPMI 1640 medium supplemented with 5% heat-inactivated autologous plasma. 1.5×10^5^ cells were seeded in each of the 24 wells and were cultured for 14 days to allow differentiation. The medium was then replaced with serum-free medium 1 day prior to infection. Primary macrophages were seeded in 24-well plates with or without 100 mm coverslips and were infected at an m.o.i. of 0.01 or 2, respectively. Supernatants were collected at 1, 24, 48 and 72 hpi from cells infected at an m.o.i. of 0.01 and were used to determine the replication efficiency via TCID_50_ assay. As for m.o.i. of 2, cell lysates were collected at 24 hpi by adding RLT buffer with beta-mercaptoethanol. At 24 hpi, the coverslips were fixed with 4% paraformaldehyde for immunostaining after the removal of the medium.

### Thermal inactivation assay

The kinetics of thermal inactivation of the viruses were performed by adding 1 ml of virus inoculum (with an input of 5e4, 5e3 and 5e2 TCID_50_/ml) into 24-well plates in the absence of cells as duplicates. The plates were then incubated at 37 °C, and 130 µl of supernatant was collected from each of the wells at 1, 24, 48 and 72 hpi. The virus viability was measured by TCID_50_ assay using MDCK cells.

### Desialylation–haemagglutination assay

The effect of desialylation on IAV haemagglutination of Turkey red blood cells (TRBCs) was studied as previously described [21,22]. TRBCs (Lampire) in Alsevers were washed and diluted to 0.5% in 1× PBS at pH 7.4 without Ca^2+^ or Mg^2+^. They were treated with either 20 mU ml^−1^ Glyko® Sialidase S™ (Prozyme), 200 mU ml^−1^ Glyko® Sialidase C™ (Prozyme) or 100 U ml^−1^ DAS181 (NexBio Inc) for 2 h at 37 °C. After the treatment, TRBCs were washed with 1× PBS, centrifuged at 2,000 r.p.m. for 5 min and repeatedly washed until the supernatants become clear. The pellet was then resuspended in 1× PBS to 0.5%. Viruses were twofold serially diluted with 1× PBS in Greiner CELLSTAR® Clear V-bottom 96-well plates (Sigma-Aldrich) in triplicate. Equal volumes of the treated or untreated 0.5% TRBCs were subsequently added to 50 µl of the diluted viruses/1× PBS (as negative control) in each of the wells. The plates were then incubated at room temperature for 20 min, and the haemagglutination titres were calculated as the reciprocal of the highest dilution that gave haemagglutination.

### TCID_50_ assay

Confluent 96-well MDCK cell plates were seeded 1 day before use. Cells were washed once with 1× PBS at pH 7.4 without Ca^2+^ or Mg^2+^ and replenished with Minimum Essential Medium (Gibco) supplemented with 100 U ml^−1^ penicillin-streptomycin (Gibco), 0.025 M HEPES (Gibco) and 1 µg ml^−1^ Tosyl phenylalanyl chloromethyl ketone-treated trypsin from bovine pancreas (Sigma-Aldrich). The virus was serially diluted at half-log10 dilution depending on the hpi and added to the cell plates in quadruplicate. Plates were incubated in an incubator at 37 °C with 5% CO_2_ until 72 hpi and were used to obtain the viral dilution endpoints. The Karber method was used to determine the viral dilution endpoints, leading to cytopathic effect in 50% of inoculated wells.

### Immunofluorescence staining

Primary macrophages that were seeded on coverslips were fixed at 24 hpi using 4% paraformaldehyde for immunofluorescence staining. In brief, 0.1% Triton-X with PBS was used to permeabilize the cell membrane for 45 min at room temperature on a shaker. The coverslips were washed with 1× PBS, followed by incubation with Fc blocker for 20 min with mild shaking. The cells were stained with mouse anti-influenza virus nucleoprotein and matrix antibody conjugated with FITC (Dako) in the dark for 1 h at 37 °C. The cells were further incubated with 1:2,000 DAPI prior to washing with 1× PBS and water before mounting. A temporary mounting medium made up of PBS and glycerol was used as the mounting medium.

### Immunohistochemistry staining

The human tissues for *ex vivo* infection were fixed at 72 hpi with 10% formalin. The fixed tissues were then embedded in paraffin and sliced into 4 µm sections. The sections were subsequently microwaved for 15 min for antigen retrieval, followed by quenching with 3% H_2_O_2_ for 20 min to stop the endogenous peroxidase activity. The slides were blocked with 10% normal horse serum at room temperature and incubated with the primary NP-specific mouse monoclonal antibody (HB65) for 90 min, followed by peroxidase (HRP)-conjugated anti-mouse antibody to stain for influenza A virus nucleoprotein. The cell nuclei were counterstained with Mayer’s haematoxylin, and the sections were developed under NovaRED substrate Kit.

### Quantification of cytokine and chemokine gene expression

RNA was extracted from cell lysates collected at 24 hpi using MiniBEST universal RNA extraction kit (Takara) and reverse transcribed using PrimeScript RT reagent Kit (Takara). The mRNA expression was quantified using real-time PCR amplification with SYBR green (Takara) and an ABI ViiA™ 7 real-time PCR system (Applied Biosystems) according to the manufacturers’ instructions. Absolute mRNA copy numbers of *β*-actin (forward primer: 5′-TGGATCAGCAAGCAGGAGTATG-3′ and reverse primer: 5′-GCATTTGCGGTGGACGAT-3′), IAV matrix (M) gene (forward primer: 5′-GGCATTTTGGACAAAKCGTCTA-3′ and reverse primer: 5′-CTTCTAACCGAGGTCGAAACG-3′), IFN-*β* (forward primer: 5′-CAACTTGCTTGGATTCCTACAAAG-3′ and reverse primer: 5′-TGCCACAGGAGCTTCTGACA-3′), IL-29 (forward primer: 5′-GCCCCCAAAAAGGAGTCCG-3′ and reverse primer: 5′-AGGTTCCCATCGGCCACATA-3′), TNF-*α* (forward primer: 5′-GCAGGTCTACTTTGGGATCATTG-3′ and reverse primer: 5′-GCGTTTGGGAAGGTTGGA-3′), monocyte chemoattractant protein-1 (MCP-1) (forward primer: 5′-CAAGCAGAAGTGGGTTCAGGAT-3′ and reverse primer: 5′-TCTTCGGAGTTTGGGTTTGC-3′), IL-6 (forward primer: 5′-GCATGGGCACCTCAGATTGT-3′ and reverse primer: 5′-TGCCCAGTGGACAGGTTTCT-3′), RANTES (forward primer: 5′-CTTTGCCAGGGCTCTGTGA-3′ and reverse primer: 5′-GCAGTGTTCCTCCCCTCCTT-3′), IFN-stimulated gene 15 (ISG15) (forward primer: 5′-CAAATGCGACGAACCTCTGA-3′ and reverse primer: 5′-CCGCTCACTTGCTGCTTCA-3′), IP-10 or CXCL10 C-X-C motif chemokine 10 (CXCL10) (forward primer: 5′-ATTATTCCTGCAAGCCAATTTTG-3′ and reverse primer: 5′-TCACCCTTCTTTTTCATTGTAGCA-3′), IFN-induced GTP-binding protein Mx1 (MX1) (forward primer: 5′-GAGGCCAGCAAGCGCAT-3′ and reverse primer: 5′-TGGAGCATGAAGAACTGGATGA-3′) and macrophage inflammatory protein 1 beta (forward primer: 5′-CACCGCCTGCTGCTTTTC-3′ and reverse primer: 5′-TAATCTACCACAAAGTTGCGAGGAA-3′) were determined with standard curve method [21,22]. The mRNA expression of all genes was normalized to that of *β*-actin.

### Sequencing analysis

Sequence alignment and analysis were performed using Geneious Basic (v2021.1.1). The sequences of reference swine H1N1 viruses were randomly selected from the GenBank or GISAID database. nt sequences of eight genes of swine influenza A viruses were aligned using MAFFT v7.490 [23]. Maximum-likelihood trees inferred from nt sequence alignment were constructed using RAxML MPI v8.2.12 [23] with GTRGAMMA applied as the nt substitution model. Data were bootstrap resampled 1,000 times. Phylogenetic trees were visualized using the ggtree package [24] in R.

### Statistical analysis

Data analysis and statistical analysis were performed using GraphPad Prism v9. All graphs were plotted as averages with sd (mean±sd). At least three independent sets of experiments were performed in both *in vitro* and *ex vivo* studies, and each infection was performed as duplicates whenever applicable.

One-way ANOVA was used to compare the cytokine and chemokine mRNA expression levels using Tukey’s multiple comparison test. Area under the curve (AUC) values were calculated by the areas under the replication kinetic curves from 24 to 72 hpi following the trapezoid rule [25]. Two-way ANOVA with Tukey’s multiple comparison test was used to compare the viral titre between viruses at each time point. The detection limit of the TCID_50_ assay was 1.456 TCID_50_/ml. Data with *P*<0.05 are considered of statistical significance.

## Results

### Receptor binding

In order to investigate the receptor-binding specificities of G4 EA H1N1, we sought to use untreated and desialylated 0.5% TRBCs, respectively, to perform haemagglutination on serially diluted viruses. The H1N1/pdm/09 strain was used as a control as it was known to be predominantly binding to α-2,6-linked sialic acid [26,27]. The treatment using sialidase S, which would cleave α-2,3-linked unbranched sialic acid, has no effect on preventing the haemagglutination of TRBCs on SW13, SW2885, H1N1/pdm/09 and G1. Meanwhile, the treatment using sialidase C, which cleaves both α-2,3- and α-2,6-linked unbranched sialic acid, has resulted in a 2–32-fold decrease of haemagglutination on all four viruses (Table 1). These data suggest that G4 EA H1N1 viruses have a major binding affinity to human influenza virus receptor with α2,6 sialic acid linkage.

**Table 1.** Desialylation effect on virus haemagglutination of TRBCs

Virus	0.5% TRBC		
	Untreated	Sialidase S™	Sialidase C™
SW13	128	128	8
SW2885	128	128	16
H1N1/pdm/09	128	128	4
G1	256	256	128

Haemagglutination titres were calculated as the reciprocal of the highest dilution that gave haemagglutination. Identical results were obtained from three independent experiments.

### Sequence analysis

In order to obtain a better understanding of the molecular features of the two G4 EA H1N1 swine influenza associated with viral pathogenicity, transmissibility and antiviral resistance (Table 2), we sought to perform sequence analysis on SW13 and SW2885 using H1N1/pdm/09 as references. The HA gene of SW13 and SW2885 viruses has unveiled differences in the aa residue of the glycosylation motif and in several receptor-binding sites. While in PB2, the well-known mutations E627K [28,29] and D701N [28,30] were not detected in either SW13 or SW2885, whereas the mutation T588I was observed in both viruses [31]. The NA H275Y mutation [32,33], which has been reported globally for developing oseltamivir resistance, was not seen in SW13 nor SW2885. Another frequently reported S31N mutation in the M2 gene, which was responsible for amantadine resistance [34], was as well not observed in either SW13 or SW2885.

**Table 2.** Molecular features associated with viral pathogenicity, transmissibility and antiviral resistance

Virus	HA							na		PB2				
	Cleavge Site	Glycosylation motif	Receptor-binding site											
		172–174#	201	207	210	147–151	239–242	275+	295	588	590	591	627	701
SW13	IQSRG	GNS	T	T	Q	RGTTV	EQAG	H	N	I	S	R	E	D
SW2885	IQSRG	GNS	T	T	Q	RGTTV	EQAG	H	N	I	S	R	E	D
H1N1pdm	IQSRG	ENS	T	S	Q	KGVTA	DQEG	H	N	T	S	R	E	D
G1	RSSRG	SGF	T	N	I	TGISR	DLQG	H	N	A	S	K	E	D

HA: haemagglutinin; NA: neuraminidase; PB2: polymerase basic 2; M2: matrix-2; PA: polymerase acidic; +: molecular markers of oseltamivir-resistance.

Since the G4 strains we used in the study were isolated ~10 years ago, we therefore performed a phylogenetic analysis to compare with strains from 2004 to 2020 (details of strains being used for phylogenetic analysis could be found in Table S1, available in the online Supplementary Material). All eight segments were aligned and compared at nt level to see if there would be any significant genetic differences (Fig. S1). It was shown that in the HA gene, the two G4 strains are still classified in the same clade (clade 1.C.2.3), same as other more recent strains. In terms of other segments, both G4 strains used in this study have the same root compared with other more recently isolated G4 strains, indicating that there were no significant genetic differences.

### Replication competence of swine G4 influenza virus in *ex vivo* human bronchial and lung explants

**Fig. 1.**
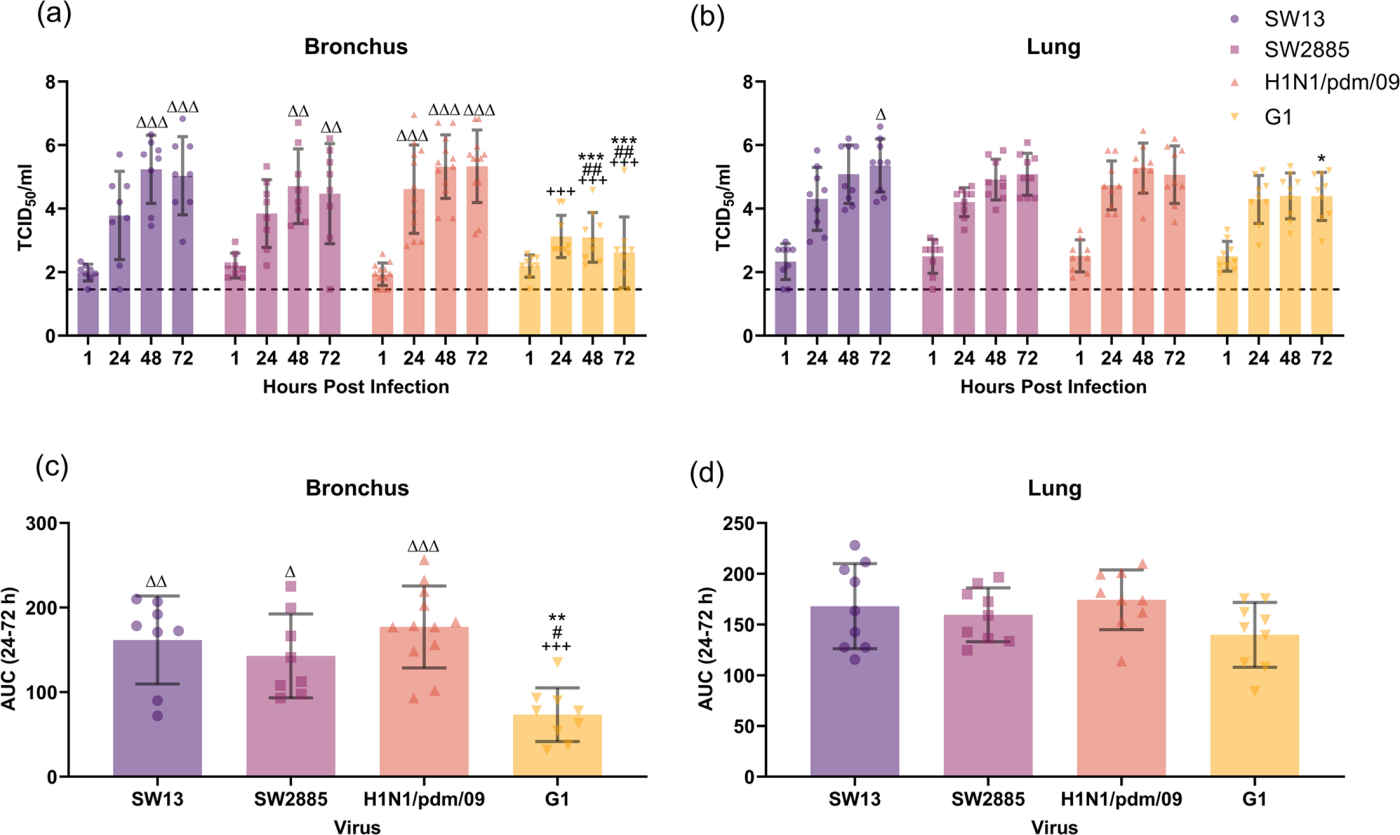
Viral replication kinetics of SW13, SW2885, H1N1/pdm/09 and G1 in *ex vivo* culture of human (**a**) bronchus and (**b**) lung with 106 TCID_50_/ml virus at 37 °C. The viral titre in the culture supernatant was determined by TCID_50_ assay, with a detection limit of 1.456 TCID_50_/ml. The data were pooled from at least three independent experiments and shown as mean±sd. Two-way ANOVA with Tukey’s multiple comparison test was used to compare the viral titre between viruses at each time point. The AUC over 24–72 hpi is shown for both (**c**) bronchus and (**d**) lung. The data were pooled from at least three independent experiments and shown as mean±sd. Statistical analysis was calculated by one-way ANOVA. *, *P*=0.033; **, *P*=0.002; and ***, *P*<0.001 (compared to SW13). #, *P*=0.033; ##, *P*=0.002; and ###, *P*<0.001 (compared to SW2885). +, *P*=0.033; ++, *P*=0.002; and +++, *P*<0.001 (compared to H1N1/pdm/09). Δ, *P*=0.033; Δ Δ, *P*=0.002; and Δ Δ Δ, *P*<0.001 (compared to G1).

We have determined the infectivity and viral replication competence of the two G4 strains, SW13 and SW2885, with H1N1/pdm/09 and G1 in human bronchial and lung *ex vivo* explants. All the above viruses used have demonstrated productive replication in *ex vivo* bronchial explants. SW13 has demonstrated a replication with a 2 log10 increase in TCID_50_ from 24 to 72 hpi. The replication pattern of SW13 is similar to that of H1N1/pdm/09 at most time points, peaked at 48 to 72 hpi, reaching the highest titre at 10^5.2^ TCID_50_/ml. For SW2885, a lower replication efficiency was observed compared to SW13 and H1N1/pdm/09. G1 remained at relatively low levels compared with the other three viruses at ≤103 TCID_50_/ml (Fig. 1a).

Replication of the influenza viruses was also observed in *ex vivo* lung explants (Fig. 1b). Influenza viruses SW13, SW2885 and H1N1/pdm/09 have demonstrated a similar replication trend, while G1 has the lowest replication competence among all. H1N1/pdm/09 has the highest titre at 48 hpi, while SW13 has achieved the highest viral titre at 72 hpi in the lung, reaching 10^5.2^ TCID_50_/ml and 10^5^ TCID_50_/ml, respectively.

Thermal inactivation assay was performed using the four virus strains to assess the overall virus replication with the AUC analysis (Fig. S2). The AUCs calculated from 24 to 72 hpi above the detection limit of each virus were compared in between the tissue used. The AUC value for SW13 in bronchial explants was the highest among all four viruses, followed by H1N1/pdm/09. G1 has a significantly low AUC value compared with the other three viruses. In lung explants, the AUC value of H1N1/pdm/09 was the highest among all, and the trend observed has agreed with the viral replication kinetics as shown above (Fig. 1b).

### Tissue tropism of G4 EA H1N1 in *ex vivo* human bronchial and lung explants

**Fig. 2.**
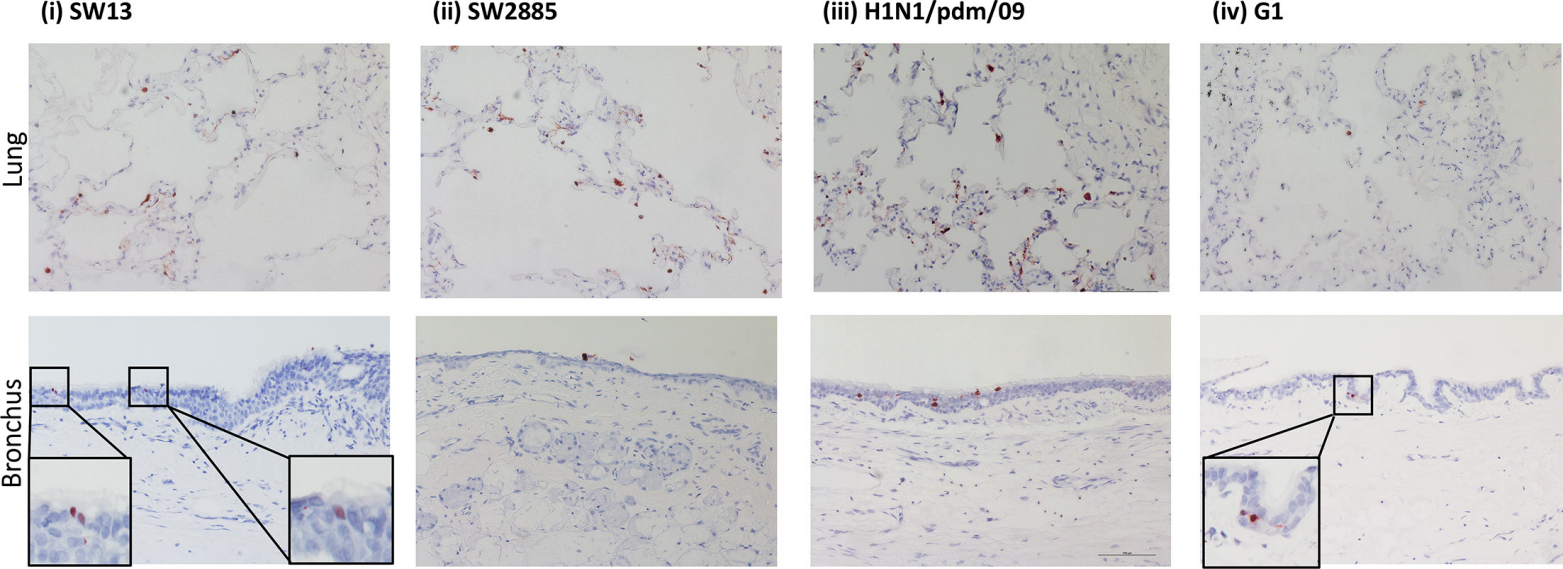
Tissue tropism of SW13, SW2885, H1N1/pdm/09 and G1 in *ex vivo* cultures of human lung and bronchus fixed at 72 hpi. Ten per cent formalin-fixed, paraffin-embedded sections of human bronchus and lung were subjected to immunohistochemical staining with a monoclonal antibody against the influenza nucleoprotein with positive cells identified by red colour. All images were taken at 200× magnification and were representative of three individual donors.

Immunohistochemical staining of the human lung tissues has demonstrated an extensive level of infection of H1N1/pdm/09 and SW13. Moderate levels of infection were observed in tissues infected by SW2885 and G1 (Fig. 2, upper panel). In the staining of human bronchial tissues, only a small amount of positively stained virus antigen cells was found in the samples infected with SW2885 and G1 (Fig. 2, lower panel). Both H1N1/pdm/09 and SW13 have illustrated a more extensive infection in the bronchus compared with those infected with SW2885 or G1. The majority of the positively stained cells were found in epithelial cells of both human lung and bronchial tissues.

### Viral replication kinetics in primary macrophage

**Fig. 3.**
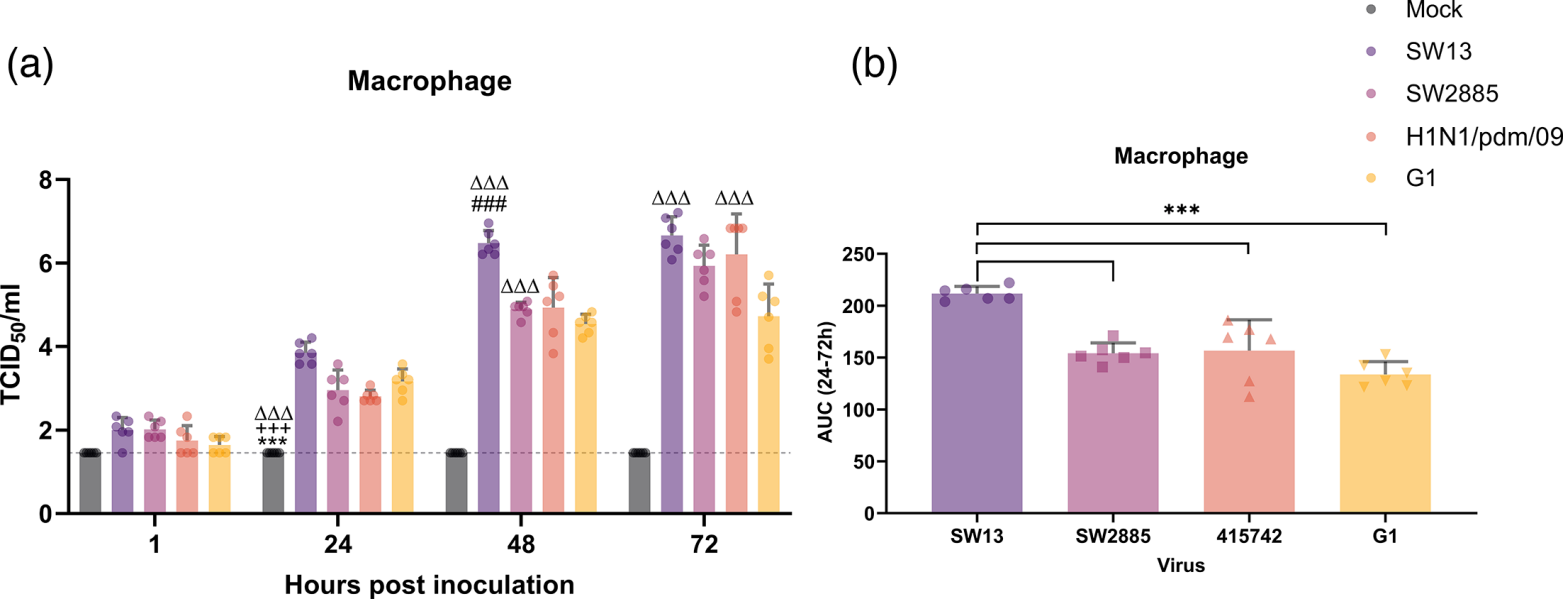
Viral replication kinetics of the G4 strains, H1N1/pdm/09 and G1 in Peripheral blood mononuclear cell (PBMC)-derived macrophages at an m.o.i. of 0.01. (**a**) The viral titre in the culture supernatant was determined by TCID_50_ assay, with a detection limit of 1.456 TCID_50_/ml. The data were pooled from three independent experiments and shown as mean±sd. Two-way ANOVA with Tukey’s multiple comparison test was used to compare the viral titre between viruses at each time point. The AUC over 24–72 hpi is shown for macrophages. (**b**) The data were pooled from three independent experiments and shown as mean±sd. Statistical analysis was calculated by one-way ANOVA. *, *P*=0.033; **, *P*=0.002; and ***, *P*<0.001 (compared to SW13). #, *P*=0.033; ##, *P*=0.002; and ###, *P*<0.001 (compared to SW2885). +, *P*=0.033; ++, *P*=0.002; and +++, *P*<0.001 (compared to H1N1/pdm/09). Δ, *P*=0.033; Δ Δ, *P*=0.002; and Δ Δ Δ, *P*<0.001 (compared to G1).

**Fig. 4.**
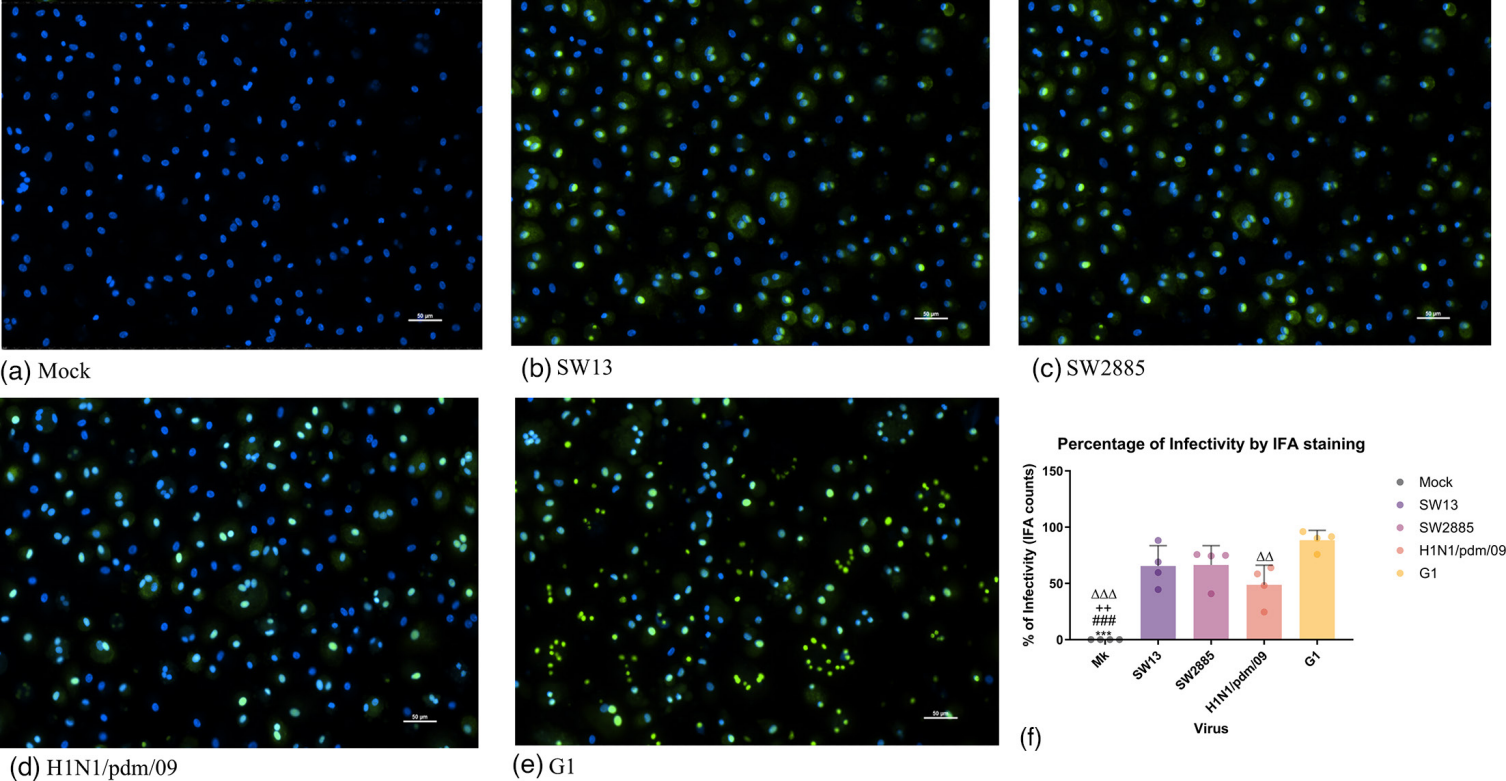
Representative immunofluorescent staining for (**a**) mock-, (**b**) SW13-, (**c**) SW2885-, (**d**) H1N1/pdm/09- and (**e**) G1 infected macrophage and (**f**) percentage of infectivity, respectively. Primary macrophages seeded on coverslips were fixed at 24 hpi using 4% paraformaldehyde for immunofluorescence staining. The immunofluorescence-stained influenza virus nucleoprotein and matrix antibody-positive cells were stained with FITC and counterstained with DAPI for macrophage nucleus. Viral infectivity was determined by counting the percentage of positive cells detected divided by the total number of macrophages (**f**) using the cell scoring function in the MetaMorph Application. The data were pooled from three independent experiments and shown as mean±sd. Statistical analysis was calculated by one-way ANOVA. *, *P*=0.033; **, *P*=0.002; and ***, *P*<0.001 (compared to SW13). #, *P*=0.033; ##, *P*=0.002; and ###, *P*<0.001 (compared to SW2885). +, *P*=0.033; ++, *P*=0.002; and +++, *P*<0.001 (compared to H1N1/pdm/09). Δ, *P*=0.033; Δ Δ, *P*=0.002; and Δ Δ Δ, *P*<0.001 (compared to G1.H).

We further studied the replication competence and innate host immune responses in *in vitro* culture of human peripheral blood-derived macrophages. Influenza virus SW13 replicated significantly higher than all four viruses, with up to 4.6 log10 increase of viral titre at 72 hpi. Meanwhile, H1N1/pdm/09 has demonstrated an increase in viral titre of 1.1 to 4.5 log10 from 1 to 72 hpi. A similar trend of viral replication was observed in both SW2885 and G1, with an increase of viral titre of 3 to 3.1 log10 (Fig. 3a). The AUC values obtained have exhibited the same trend as the TCID_50_ assay showed (Fig. 3b). The immunofluorescence staining further confirmed the presence of viral protein in macrophage upon infection (Fig. 4a–e). All the influenza viruses used have infected >48% of primary macrophage (at an m.o.i. of 2) by 24 hpi, as shown by immunofluorescence staining (Fig. 4f). The percentages of infectivity of SW13 and SW2885 were 65% and 66%, respectively. Immunofluorescence staining corresponds to TCID_50_ results as no significant difference was found between the two G4 strains SW13 and SW2885. A significant difference was found between H1N1/09 and G1. G1 virus (88%) had the highest percentage of infectivity, while H1N1/09 (48%) showed the lowest percentage of infectivity out of the four virus strains in primary human macrophage.

### Influenza G4 virus induces a mild cytokine and IFN responses in macrophage

**Fig. 5.**
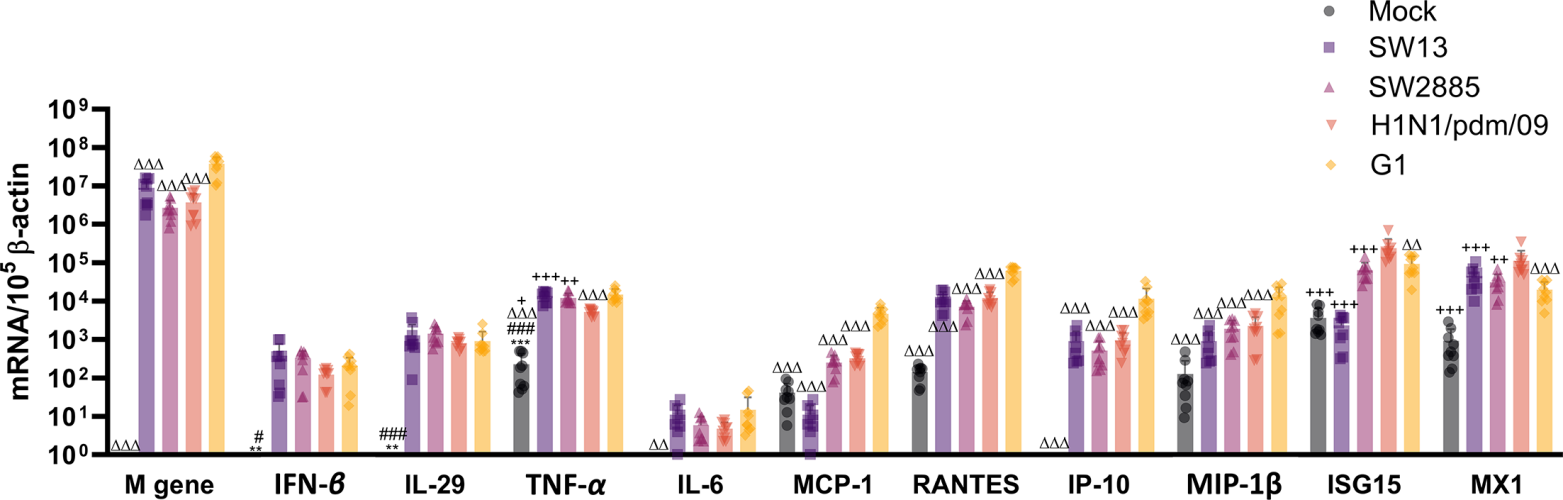
Cytokine and chemokine mRNA expression profile in PBMC-derived macrophages infected at an m.o.i. of 2 with mock, SW13, SW2885, H1N1/pdm/09 and G1. The expression of the mean mRNA copies per 10^5^ *β*-actin copies of influenza matrix (M) gene, IL-29, TNF-*α*, IFN-*β*, IL-6, ISG15, MCP-1, MX1, RANTES, IP-10 and MIP-1*β* obtained at 24 hpi. The mean was calculated based on the data pooled from four independent experiments and presented as mean±sd. Statistical analysis was calculated by one-way ANOVA. *, *P*=0.033; **, *P*=0.002; and ***, *P*<0.001 (compared to SW13). #, *P*=0.033; ##, *P*=0.002; and ###, *P*<0.001 (compared to SW2885). +, *P*=0.033; ++, *P*=0.002; and +++, *P*<0.001 (compared to H1N1/pdm/09). Δ, *P*=0.033; Δ Δ, *P*=0.002; and Δ Δ Δ, *P*<0.001 (compared to G1).

A comparable level of influenza matrix gene (M gene) mRNA expression was observed in SW13-, SW2885-, H1N1/pdm/09- and G1 infected macrophage at 24 hpi. G1 and H1N1/pdm/09 served as the control of high and low cytokine-inducing virus, respectively, for comparison. G1 induced a significantly high induction of chemokines (MCP-1, RANTES, IP-10 and MIP-1*β*) upon infection than SW13, SW2885 and H1N1/pdm/09. Both SW13-, SW2885- and H1N1/pdm/09-infected macrophages have resulted in a relatively weak induction of IFN-*β*, IL-29 and IL-6 at 24 hpi. The G4 EA H1N1 viruses (SW13 and SW2885) have induced a less intense secretion of IFN-stimulated genes, MX-1 and ISG-15, compared with H1N1/pdm/09 (Fig. 5).

## Discussion

Here, we evaluated the viral replication competence, tissue tropism and innate host responses of the G4 EA H1N1 swine influenza in a highly physiologically relevant platform of the *ex vivo* explant culture of the human bronchus and lung and in *in vitro* culture of human peripheral blood-derived macrophages. We have demonstrated that the G4 EA H1N1 swine influenza can replicate effectively in both human lung and bronchial explants and in PBMC-derived macrophages. The G4 EA H1N1 swine influenza virus is a mild cytokine inducer upon infection.

Viral tropism is of enormous importance in terms of investigating the transmission and pathogenesis of a virus. The ability for an influenza virus to effectively infect human conducting airways (trachea and bronchi) has been listed by the World Health Organization as one of the risk assessment platforms [35,36] for assessing the viral transmissibility and correlates consistently with *in vivo* ferret transmission study [37]. One of the pre-requisites for influenza viruses to acquire efficient human-to-human transmission is to be able to replicate effectively in the upper and conducting airways [36,38]. We have shown that in *ex vivo* bronchial explants, the two G4 strains could replicate effectively, to a similar competence with H1N1/09 pandemic and significantly more competent than the avian G1 virus. Although G1 was an avian influenza virus, the G1 lineage has been proven to be able to replicate in mammalian tissues [39]. Also, many of the H9N2 viruses have demonstrated the capacity in binding both α-2,3 and α-2,6 receptors, predominantly to α-2,6 receptors [40]. The haemagglutination results have further illustrated that G4 viruses preferentially bind to the human-like α2,6 linkage receptor, which was also reported by the group that revealed the G4 viruses [1]. It has been demonstrated by a couple of studies that G4 EA H1N1 is highly transmissible to ferrets by either direct contact or airborne droplets [1,41, 42], posing a greater threat towards human transmission. The ability of G4 EA H1N1 to cause infection in different animals has enhanced the opportunity for better adaptation in human and may generate future pandemic strains [43].

The ability of the influenza virus to infect the lung is proven to be correlated with the disease severity [44,45]. It has been previously shown that viruses that infect the alveolar epithelium (lower lung) are less transmissible and are more likely to result in severe viral pneumonia in human, whereas those that replicate better in conducting airway are the opposite [38]. For the two G4 strains, other than replicating productively in *ex vivo* bronchial explants, we have also revealed their ability to replicate effectively in human lung explants. The replication competence of the two G4 strains is similar to H1N1/09 pandemic and G1. The trend of generally high viral replication competence in human lung may indicate that the G4 virus can productively replicate in human lung. A number of human cases caused by EA H1N1 have been reported in Europe and Asia, including the Netherlands, Spain, Italy, Switzerland and China since 2013, indicating an increase in human acquiring EA H1N1 infection. Human cases caused by EA H1N1 have been reported occasionally in Europe and Asia since 2003 [2,6,46]. Fortunately, these cases are still limited to people who are involved in the swine industry. Other than being able to infect human lung and bronchus, population immunity is another common way to determine the pandemic potential of influenza strains. It has been reported that G4 EA H1N1 strains reacted poorly with the currently used H1N1 vaccine by either using ferret’s sera or human serum sample, indicating that the G4 EA H1N1 strains may pose a pandemic risk [42,49].

Furthermore, we showed that G4 virus is a mild inducer of pro-inflammatory cytokine in human macrophages, a major cell type that induces cytokine and chemokine upon influenza virus infection and was shown to play a major role in inducing cytokine storm [20]. We have shown that the primary culture of human macrophages was susceptible towards all four strains of influenza viruses and thus a comparable induced host innate immune response between the swine G4 virus and the H1N1pdm virus-infected macrophages. The mRNA expression of IL-29, TNF-*α* and IFN-*β* for the G4 swine viruses were similar to the level achieved in G1, whereas the expressions of ISG15 and MX1 were particularly high in H1N1/pdm/09 infection. This difference may suggest that they have triggered different pathways of IFN activation upon virus infection. The pro-inflammatory cytokine and chemokine responses induced by the G4 strains were similar to H1N1/pdm/09, indicating that they were less potent inducers compared with G1.

Finally, through complete genomic sequence analysis, we sought to compare the two EA G4 H1N1 swine influenza with the well-documented globally circulated H1N1/09 pandemic strain to investigate the viral pathogenicity, transmissibility and antiviral resistance. We have not observed any of the mutations that have been widely reported in the HA, NA, PB2 and M2 genes. We have also found mutations at 590S/591R in the PB2 protein, these mutations have been previously reported to enhance the pathogenicity of 2009/H1N1 virus in mammals [42]. This SR polymorphism may represent an additional strategy to help with influenza to reduce the selective pressure over E627K mutation in PB2 and the high prevalence of 590S/591R in viruses isolated from swine indicated that these viruses are partially adapted for human replication [50].

Taken together the effective replication competence of the two G4 strains in both human lung and bronchial explants, we can therefore extrapolate that there is a potential risk of increasing human transmission. The cytokine induction upon infection has demonstrated possibly mild disease severity in human. The two mutations, T588I and 590S/591R, both identified in PB2 of the G4 strains, may favour the adaptation of G4 EA H1N1 strain for human infection and potentially lead to future pandemics. It is of concern that the G4 EA H1N1 virus may pose a potential public health threat, given they have acquired many of the essential capabilities to be able to infect human conducting and lower airway. Therefore, the risk assessment of G4 in the human respiratory tract remains important. A more physiologically relevant risk assessment platform should apply to validate the data acquired using cell lines and animals (mice and ferrets) to assess the G4 EA H1N1 strains. Routine surveillance and close monitoring of the swine industry may help to prepare us for the noticeable threat.

## Supplementary material

10.1099/jgv.0.002133 Uncited Supplementary Material 1.

## Abbreviations

AUC: area under the curve
EA: Eurasian avian-like
G4: genotype 4
hpi: hours post infection
ISG15: IFN-stimulated gene 15
MCP-1: monocyte chemoattractant protein-1
MDCK: Madin–Darby canine kidney
TR: triple reassortant
TRBC: Turkey red blood cell
