# Supplementary material for: Evaluation of pandemic potential of the genotype 4 (G4) swine influenza virus using ex vivo and in vitro cultures of the human respiratory tract
Source: J Gen Virol. 2025 Jul 31;106(7):002133. doi: 10.1099/jgv.0.002133 (PMC12313140; doi:10.1099/jgv.0.002133)
Supplement: Uncited Supplementary Material 1. [file jgv-106-02133-s001.pdf]

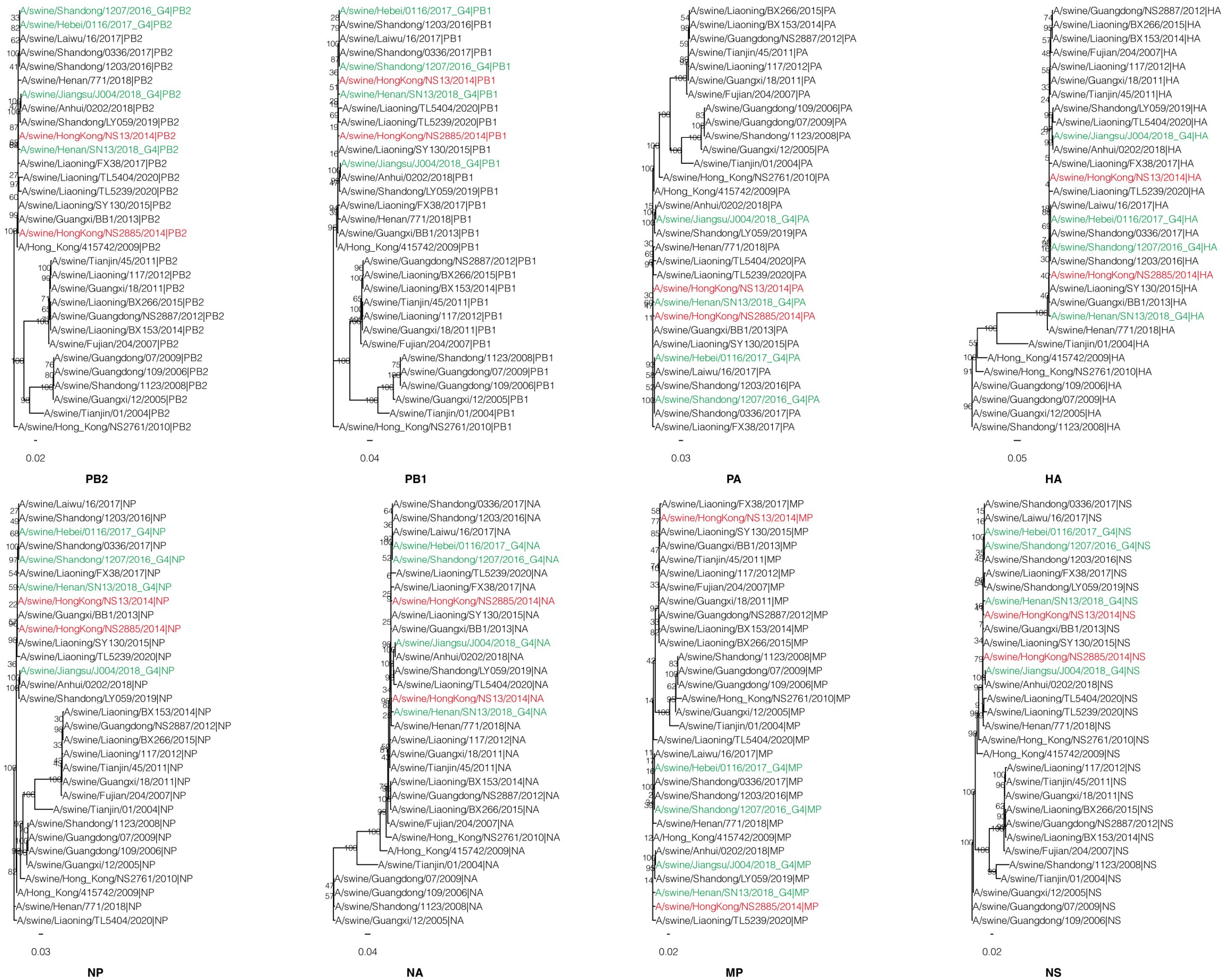

Supplementary Figure 1. Maximum-likelihood phylogenetic tree of eight genes of swine influenza A viruses (H1N1) from pigs circulating in China. The sequences of related viruses were randomly selected from GenBank or GISAID database. The maximum-likelihood trees were constructed using RAxML MPI v8.2.11 (23) based on the nucleotide sequence alignment of eight genes of indicated H1N1 variants respectively. The four reference viruses in lineage G4 (clade 1.C.2.3) were highlighted with green, and two interested viruses isolated from Hong Kong were highlighted with red.

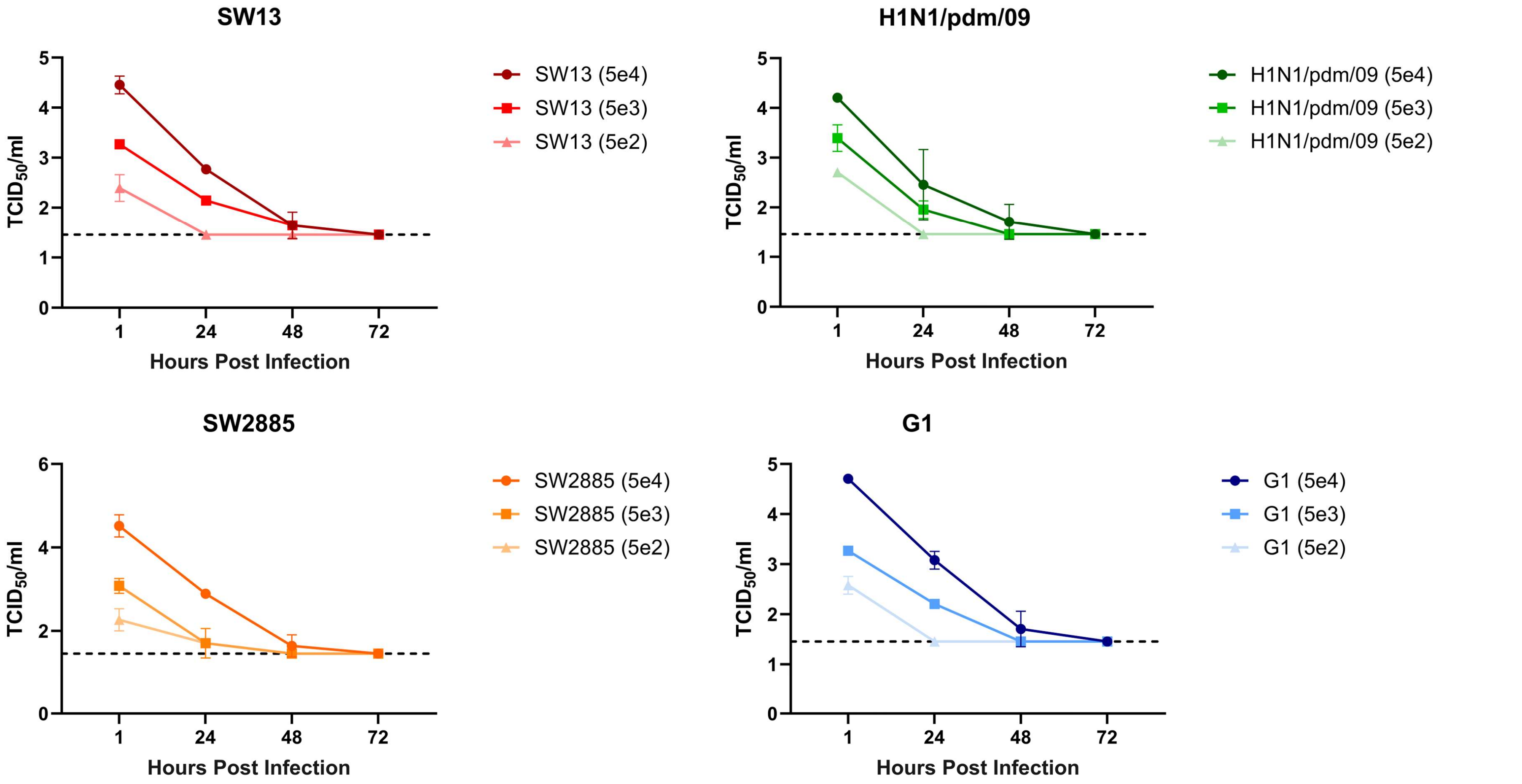

Supplementary Figure 2. Thermal inactivation assay of SW13, SW2885, H1N1/pdm/09 and G1. The kinetics of thermal inactivation of the viruses were performed by adding 1 ml of virus inoculum (with an input of 5 x $10e^4$, 5 x $10e^3$ and 5 x $10e^2$ TCID50/ml) into 24 well plates in the absence of cells as duplicates. The plates were then incubated at 37°C and 130 µl of supernatant was collected from each of the wells at 1, 24, 48 and 72 hours post inoculation. The virus viability was measured by $TCID_{50}$ assay using MDCK cells.

TableS1. Accession numbers swine H1N1 used in phylogenetic analysis.

Name	Year of Isolation	Isolate ID		Seq ID							
		GISAID	NCBI (HA)	PB2	PB1	PA	HA	NP	NA	MP	NS
A/swine/Liaoning/TL5239/2020	2020		OL310843	OL310970	OL311363	OL311396	OL310843	OL311011	OL310945	OL314773	OL311135
A/swine/Liaoning/TL5404/2020	2020		OL310844	OL310987	OL311378	OL311395	OL310844	OL311012	OL310946	OL314774	OL311136
A/swine/Shandong/LY059/2019	2019		MW127000	MW126997	MW126998	MW126999	MW127000	MW127001	MW127002	MW127003	MW127004
A/swine/Anhui/0202/2018	2018	EPI_ISL_482219		EPI1751151	EPI1751224	EPI1751297	EPI1751370	EPI1751589	EPI1751443	EPI1751662	EPI1751516
A/swine/Laiwu/16/2017	2017		MK228094	MK228091	MK228092	MK228093	MK228094	MK228095	MK228096	MK228097	MK228098
A/swine/Liaoning/FX38/2017	2017		OR984415	OR984412	OR984413	OR984414	OR984415	OR984416	OR984417	OR984418	OR984419
A/swine/Shandong/0336/2017	2017		MN416640	MN416380	MN416467	MN416567	MN416640	MN418784	MN416744	MN418864	MN418710
A/swine/Shandong/1203/2016	2016		MN416642	MN416382	MN416469	MN416569	MN416642	MN418786	MN416746	MN418866	MN418712
A/swine/Liaoning/BX266/2015	2015		MN393712	MN393709	MN393710	MN393711	MN393712	MN393713	MN393714	MN393715	MN393716
A/swine/Liaoning/SY130/2015	2015		MN393816	MN393813	MN393814	MN393815	MN393816	MN393817	MN393818	MN393819	MN393820
A/swine/Liaoning/BX153/2014	2014		MN393704	MN393701	MN393702	MN393703	MN393704	MN393705	MN393706	MN393707	MN393708
A/swine/Guangxi/BB1/2013	2013	EPI_ISL_156387		EPI506660	EPI506661	EPI506662	EPI506663	EPI506664	EPI506665	EPI506666	EPI506667
A/swine/Guangdong/NS2887/2012	2012		KM029759	KM029756	KM029757	KM029758	KM029759	KM029760	KM029761	KM029762	KM029763
A/swine/Liaoning/117/2012	2012		KP404364	KP404361	KP404362	KP404363	KP404364	KP404365	KP404366	KP404367	KP404368
A/swine/Guangxi/18/2011	2011	EPI_ISL_248173		EPI911832	EPI911833	EPI911834	EPI911835	EPI911836	EPI911837	EPI911838	EPI911839
A/swine/Tianjin/45/2011	2011		KP404412	KP404409	KP404410	KP404411	KP404412	KP404413	KP404414	KP404415	KP404416
A/swine/Hong Kong/NS2761/2010	2010		KM029551	KM029548	KM029549	KM029550	KM029551	KM029552	KM029553	KM029554	KM029555
A/swine/Guangdong/07/2009	2009	EPI_ISL_75781		EPI265891	EPI265898	EPI265905	EPI265939	EPI265918	EPI265925	EPI265932	EPI265912
A/swine/Shandong/1123/2008	2008		GU646030	HM176665	GU646029	GU646035	GU646030	GU646031	GU646032	GU646033	GU646034
A/swine/Guangdong/109/2006	2006	EPI_ISL_81509		EPI287526	EPI287507	EPI287488	EPI287469	EPI287450	EPI287431	EPI287412	EPI287393
A/swine/Guangxi/12/2005	2005	EPI_ISL_132837		EPI407844	EPI407843	EPI407842	EPI407837	EPI407840	EPI407839	EPI407838	EPI407841
A/Hong Kong/415742/2009	2009		GU931801	GU931811	GU931815	GU931809	GU931801	GU931813	GU931805	GU931803	GU931807
A/swine/Henan/771/2018	2018	EPI_ISL_12957978		EPI2048370	EPI2048371	EPI2048369	EPI2048373	EPI2048366	EPI2048372	EPI2048368	EPI2048367
A/swine/Fujian/204/2007	2007	EPI_ISL_33887		EPI190603	EPI190604	EPI190602	EPI190597	EPI190600	EPI190599	EPI190598	EPI190601
A/swine/Tianjin/01/2004	2004	EPI_ISL_12792		EPI119937	EPI119934	EPI119932	EPI119930	EPI119928	EPI119926	EPI119920	EPI119923
